# An EEG Neurofeedback Interactive Model for Emotional Classification of Electronic Music Compositions Considering Multi-Brain Synergistic Brain-Computer Interfaces

**DOI:** 10.3389/fpsyg.2021.799132

**Published:** 2022-01-04

**Authors:** Mingxing Liu

**Affiliations:** Music School, Shanxi Normal University, Linfen, China

**Keywords:** multi-brain collaborative brain-computer interface, EEG neural, interactive electronic music, emotion classification, brain-computer interfaces

## Abstract

This paper presents an in-depth study and analysis of the emotional classification of EEG neurofeedback interactive electronic music compositions using a multi-brain collaborative brain-computer interface (BCI). Based on previous research, this paper explores the design and performance of sound visualization in an interactive format from the perspective of visual performance design and the psychology of participating users with the help of knowledge from various disciplines such as psychology, acoustics, aesthetics, neurophysiology, and computer science. This paper proposes a specific mapping model for the conversion of sound to visual expression based on people’s perception and aesthetics of sound based on the phenomenon of audiovisual association, which provides a theoretical basis for the subsequent research. Based on the mapping transformation pattern between audio and visual, this paper investigates the realization path of interactive sound visualization, the visual expression form and its formal composition, and the aesthetic style, and forms a design expression method for the visualization of interactive sound, to benefit the practice of interactive sound visualization. In response to the problem of neglecting the real-time and dynamic nature of the brain in traditional brain network research, dynamic brain networks proposed for analyzing the EEG signals induced by long-time music appreciation. During prolonged music appreciation, the connectivity of the brain changes continuously. We used mutual information on different frequency bands of EEG signals to construct dynamic brain networks, observe changes in brain networks over time and use them for emotion recognition. We used the brain network for emotion classification and achieved an emotion recognition rate of 67.3% under four classifications, exceeding the highest recognition rate available.

## Introduction

Brain-computer interface (BCI) is a communication system that does not rely on the brain’s normal peripheral nerve and muscle output pathways. BCI enables direct communication between the human brain and the outside world or device control by creating a virtual channel between the human brain and an external device, which provides a possible path to recovery for people with paralysis or other motor dysfunction disorders (Rossetti et al., 2020). BCI technology involves neuroscience, computers, signal processing, pattern recognition, and other disciplines, and through the processing and classification of physiological signals collected from the human brain, it identifies the various intentions of the subject, and then converts the intentions into specific control commands, thus achieving direct control of external devices by the human brain. However, few BCIs systems can be put into large-scale applications. BCI technology involves neuroscience, computer, signal processing, pattern recognition and other disciplines. By processing and classifying the physiological signals collected from the human brain, it recognizes various intentions of the subjects, and then converts the intentions into specific controls. Command, so as to realize the direct control of the human brain to the external equipment. This is partly because real-time systems do not meet the requirements of large-scale applications in terms of performance, and partly because most of the current BCI systems are still stuck in the synchronization mode. Synchronous mode means that the system pre-defines the time window in which the user needs to operate, and when the system starts, the user always needs to pay attention to the system prompt and start or stop the brain control operation according to the time window prompted by the system, and the system will continuously classify and identify each time window, and perform the operation according to the classification result (Farrell, 2021). On the one hand, the user needs to focus on the system prompts all the time, which prevents the user from performing daily actions outside the system and increases the user’s mental load, which can lead to fatigue. On the other hand, the user cannot start or stop the operation at any time according to his or her wish. When the user needs to operate, the system needs to be started first. When the user does not need to operate, the system cannot stop the operation at any time according to the user’s will, but continues to continuously output commands and execute the operation until the system is shut down, which greatly destroys the autonomy of the BCI system.

In the field of art, visual art and auditory art are the most common forms of art, each of which can provide people with both physical and psychological aesthetic experiences (Dahan, 2020). In the traditional sense, auditory art can only be experienced through the auditory senses, and the form of experience is relatively single. With the development of multimedia technology, people have put forward higher requirements for the aesthetic experience of auditory art, seeking more diverse forms of artistic expression. The visualization of sound provides people with a new form of visual and auditory expression for the appreciation of auditory art, and the new visual interpretation of auditory art adds to the expressiveness of auditory art, providing people with the dual beauty of the combination of visual and auditory senses, and the visualization of sound is conducive to people’s deep understanding of the formal structure and emotional content of artworks. For example, in the live performance of the Czechia Philharmonic Orchestra, the designer graphically designed the music to be played according to the rhythm, and displayed the 3D effect simultaneously with the performance, bringing a visual feast to the audience. With the advancement of technology and different application needs, forms of interaction between humans and machines and systems are used in various fields, and interactive sound visualization is developing from the purely artistic field to more practical directions, such as aiding applications in medical, entertainment, and teaching fields (Simurra and Borges, 2021). Interactive sound visualization can play the audience’s specificity to produce different visual images, so it can be used in the diagnosis of certain articulation diseases and aided medical applications; in music teaching, interactive sound visualization can show students the visual structure of music, facilitating students to operate intuitively according to their learning situation; interactive sound visualization can play an active role for hearing-impaired people in speech recognition. Interactive sound visualization can play an active role in speech recognition, music performance, and other aspects.

Different cultures understand music differently, but they all have one thing in common and that is that through music one can experience the culture for a short period. Music is a way for composers to express their thoughts and feelings to listeners through specific sound structures and melodic variations; music portrays emotions and moods in different environments to listeners through sound (Turchet et al., 2021). The user needs to pay attention to the system always prompts, start, or stop the brain control operation according to the time window prompted by the system, the system will continuously classify and identify each time window, and perform operations according to the classification results. One of the basic cognitive functions of humans is the emotional response to music. Different people may have different emotional reactions to the same music because of their different life experiences, but music expresses the composer’s thoughts and feelings and he will infect the listener’s thoughts and feelings with a strong rendering, and from this perspective, the impact of music on different people’s thoughts and feelings is again similar. Accurate recognition of musical emotions can achieve harmonious interaction between human and machine emotions, which has great application prospects in various fields. For example, in daily life, real-time emotion recognition can help people regulate their emotional state promptly and change the current emotional state of a person through music to reduce accidents caused by emotional factors.

## Current Status of Research

The new research method of parsing musical elements and describing the changes in the corresponding quantitative values of different elements and emotions has pioneered the empirical study of musical emotions, allowing a close connection between the two. The results available so far show that emotions related to music are divided into two main aspects: object and subject (Wolffenbüttel and Pellin, 2020). The object is the listener’s perception of the emotion expressed in the musical work itself, i.e., the perception of the emotion the composer wants to express, which is universal and does not vary according to individual differences; the subject is the emotional response of the listener’s nature that is evoked by the musical stimulus (Iverson, 2019). Instead, it continues to output instructions and perform operations until the system is shut down, which greatly undermines the autonomy of the BCI system. The main difference between object and subject is that the object is universal and the subject has individual differences. The Musical Cue Coherence Model posits that brain processing of musical events is the process by which the brain extracts different musical features and then achieves integration (Turchet et al., 2021). Emotion is the brain’s representation of these different musical features, and the listener’s emotional perception can be predicted by elements of music such as key, melody, and rhythm. Major keys in classical music correspond mostly to positive emotions, while minor keys correspond mostly to negative emotions (Biasutti and Concina, 2021). The synesthesia theory of musical emotion suggests that the process of the listener’s enjoyment of music is the process of achieving interaction with the creator and that the occurrence of musical emotion is the process of achieving emotional empathy and sympathy between the listener and the composer (Malecki et al., 2020).

Synesthesia theory suggests that a person interacting with the external environment causes the person to align with the environment by adapting themselves to it (Romão, 2020). The listener’s process of listening to music is the process of aligning himself or herself with the emotions of the music. This theory emphasizes the music itself at the expense of the listener’s subjective feelings (Lakatos, 2020). The areas activated by dissonant music are more pronounced and include the hippocampus, Para hippocampal gyrus, and temporal pole which equate to areas highly relevant to emotional perception. At the same time, the arousal of the inferior frontal gyrus, and inferior Brodmann’s area was stronger for harmonic music, and the activation of the relevant parts reflected, to some extent, the operational processes of musical structure, syntactic analysis, and memory. It is noted that the activation of brain regions increases with increasing stimulation time, indicating to some extent the dynamic effect of emotion (Redhead, 2021). In conjunction with the use of ER-related event analysis in his study, it was found that the pattern of neural activation differed between the two situations, as expected and differently expected and that the brain regions and brain activity activated during listening to favorite, familiar music, or singing belonged to positive emotions. This finding is similar when complexity changes are introduced, their activation patterns, and discordant intervals are present. It is easy to see that the expression of emotions by music involves extremely complex cognitive processes. The new visual interpretation of auditory art adds to the expressive power of auditory art, and provides people with a dual aesthetic that combines audiovisual senses.

The first thing that is evoked when listening to music is a memory mechanism, where the brain integrates self-knowledge and activates autobiographical nerves. So, there is a strong emotional trigger on familiar songs. Also because of the similarity of the left-skewed spiking tract that occurs when predicting the appearance of desired music to the characteristics of positive emotions (Sofer, 2020). So, it is said that familiarity with various types of music directly influences to some extent the emotional experience it evokes. When the musical experience is sufficiently rich for non-harmonic tunes to become part of its familiarity, the negative emotional experience is reduced to some extent. To meet the demand for continuous mining and exploration of the information carried by brain neural signals and comprehensive, flexible and in-depth exploration of peak potential signals, the thesis, based on the study of the principles of peak potential signal analysis and decoding algorithms, designs a componentized algorithm module, abstractly encapsulates the algorithm module for multiple operations, realizes flexible configuration of the analysis process, and provides two analysis modes, real-time and offline mode, which meets the experimental research requirements of peak potential signal analysis.

## Analysis of EEG Neurofeedback Interactive Electronic Music Compositions With a Multi-Brain Synergistic Brain-Computer Interface for Emotional Classification

### Multi-Brain Synergistic Brain-Computer Interface EEG Neurofeedback Analysis

The brain has a powerful information processing capacity, and several functional brain regions are involved in the processing of visual and auditory information. To investigate the specific connection process and information interaction of neural connections within the brain under brain cognitive activities, it is necessary to understand the neurophysiological basis of brain structure and the basic methods of brain cognitive experiments (Görgün, 2020). The progress of neuroscience research cannot be achieved without a rigorous experimental design and a standardized experimental process to obtain more realistic and effective experimental data of EEG signals. In this experiment, we used long-time music to induce emotion in the subjects, and at the same time, we asked the subjects to fill in the emotion scale to record their emotional states and recorded the EEG signals of the subjects during the whole experiment. It is helpful for students to operate and learn intuitively according to their own learning situation. Interactive sound visualization can play an active role in speech recognition and appreciation of music performances for the hearing impaired. The corresponding cognitive patterns were derived by analyzing the EEG signals in different emotional states, exploring the relationship between music and emotion evocation, and exploring the differences in EEG characteristics in different emotional states. It is an improved adaptive algorithm using the eigenmodal decomposition to solve the Hilbert transform in the frequency band requirement that cannot be too wide. Among the requirements for the eigenmodal decomposition function (IMF) are two. The number of zero-poles is equal or at most a little different throughout the data set. At any moment, the upper envelope consisting of the very large values and the lower envelope consisting of the very small values both have zero mean, or concerning the time axis, the upper and lower envelopes are symmetrical then the division is considered good and the next level of decomposition is performed.

First, we use two algorithms to model the offline data with different data lengths (0.5–1s with 0.1s steps) in the training phase and use the “leave-one-out” method for cross-validation: we divide the offline training data with 6 frequencies and 10 blocks into 10 groups, each containing 6 frequencies, and then use one of the groups as the test set and the other nine as the training set. We divide the offline training data with 6 frequencies and 10 blocks into 10 groups, each containing 6 frequencies, and then use one group as the test set and the other 9 groups as the training set. The template data is then used to classify and identify the data for each trial of the test set to obtain the correct classification rate for a set of test sets. After ten sets of data are tested for test set traversal, the final offline recognition correct rate is obtained by superimposing and averaging the classification correct rates of each set. When the online experiment is conducted, when the online system detects the time-synchronized signal sent from the stimulation program to the EEG amplifier, 1s of data from the sampling point where the time-synchronized signal is located is intercepted and matched with the template signal of six frequencies, respectively to obtain the final decision result and perform speech feedback.


(1) $$p_{u,v}=\frac{a^{T}\,\sum_{12}b^{2}}{\sqrt{a^{T}\,\sum_{12}a^{2}}\,\sqrt{b^{T}\,\sum_{22}c^{2}}}$$

**FIGURE 1:**
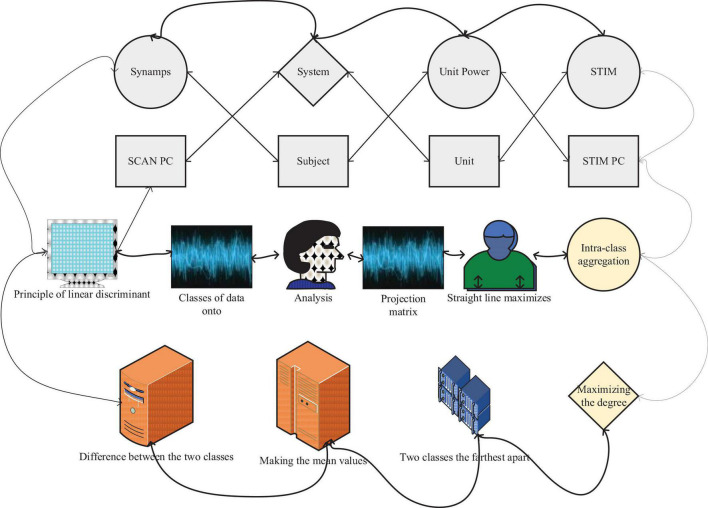
Multi-brain collaborative brain-computer interface (BCI) EEG neurofeedback framework.

Explore the differences in EEG characteristics in different emotional states. The principle of linear discriminant analysis is very simple (Smith et al., 2021). It projects two classes of data onto a straight line through a projection matrix. It is required that the projection onto the straight line maximizes the difference between the two classes of data by making the mean values of the two classes the farthest apart while maximizing the degree of intra-class aggregation in each class. The test data will be projected onto the same straight line and the class it belongs to will be decided based on the projected position. Due to its simple principle and fast computation, it is often used in the classification of EEG data, as shown in Figure 1.

EEG signals obtained by experimental paradigm evocation are subject to various noise disturbances during acquisition, as well as electrical activity caused by neural responses unrelated to the inclusion of other cognitive psychological activities, while the potential changes that are truly relevant to brain cognition are only a few microvolts, which are usually much smaller than the potential changes caused by spontaneous activity of nerve cells, so the measured EEG signals need to be preprocessed before the study of EEG signal data (D’Agostino, 2020). The common preprocessing process of the EEG technique contains steps such as EEG preview, filtering, eye-electricity removal, EEG signal segmentation, baseline correction, and conversion reference. The acquired data contains many EEG artifacts due to the accompanying blinks or eye movements of the subject during the completion of the task. The traditional method of removing the oculomotor is by placing eye electrodes, acquiring the maximum value of the oculomotor, defining the oculomotor artifacts by the percentage of the maximum value, and finally removing the oculomotor point by point. But now we can separate the oculomotor components by independent component analysis (ICA) and reduce the signal of the removed oculomotor components to achieve the effect of removing the oculomotor.


(2) $$\delta \,\left(t\right)=\sum_{k=2}^{K}I\,M_{k}\,\left(t^{2}\right)$$

(3) $$\delta_{p}\,\left(t\right)=\sum_{k=1}^{K}I\,M_{k}\,\left(t^{2}\right)/\sum_{k=1}^{M}I^{2}\,M_{k}\,\left(t\right)$$

Judgments are made after the criteria have been derived and those greater than the average contribution are recorded therein, while those less than are not calculated. After completion, the proportion of the overall that is accounted for is the one that has a particular contribution to that frequency band over a certain period and can also be considered as the dominant change. Therefore, it is further used as a parameter. The best or worst of these features are selected and given a score. After removing that feature continue the above operation with the remaining features. The result is a score of all features. It is a greedy algorithm that finds the optimal feature subspace by traversing it. One thing worth noting is that much of the stability of RFE comes from the choice of model. If a general regression algorithm is used then instability may arise, if a Ridge regression algorithm is used then Ridge itself is stable and its results are stable.

Self-encoder is an unsupervised learning neural network, whose core idea is to encode the data in the input layer (dimensionality reduction) and continuously improve the encoder and decoder by reducing the residuals between it and the original data. Ultimately, the purpose of dimensionality reduction representation is achieved. Similarly, for the input defined as a sample, supervised learning can be done by a combination of several layers of self-encoders. The method of removing electrooculogram is to obtain the maximum value of electrooculogram by placing the eye electrodes, define the electrooculogram artifact by the percentage of the maximum value, and finally remove the electrooculogram point-to-point. It is also possible to train to generate new data that is different from the samples and can be used as a generative model. Based on this the development of denoising self-encoders, stacked self-encoders, etc., is also based on the principle of reducing bias by its nature.


(4) $$\phi_{i\,j}\,\left(x\right)=\frac{1}{\delta^{d}}\,\exp\left(\frac{\left(x-x_{i\,j}\right)\,{\left(x+x_{i\,j}\right)}^{T}}{\delta^{2}}\right)$$

(5) $$E\,n\,t\,r\,o\,p\,y=\sum_{i=1}^{n}p_{i}\cdot \ln\left(p_{i}\right)$$

**FIGURE 2:**
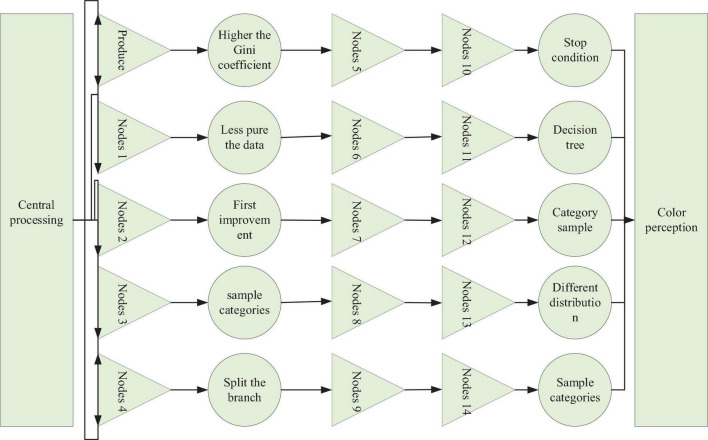
Schematic of the color vision mechanism.

As with the Shannon first, the higher the Gini coefficient means the less pure the data is based on the information first improvement there is also the use of information gain rate to overcome the problem that the number of sample categories varies and the decision tree is biased to split the branch with the most samples, as well as splitting stop conditions, pruning and other conditions not detailed here (Kerékfy, 2020). However, through the decision tree, we can find that the decision tree is also divided based on the category samples. This means that different distribution of sample categories may produce different tree nodes. As an anisotropic classification, decision trees also meet the requirements of music emotion model learning well but face the same huge dimensionality problem. Decision trees can be combined to obtain an optimal feature subspace, as shown in Figure 2. The physiological characteristics of the EEG signal representations vary with frequency, so the EEG signals in different frequency domains differ in their discrimination between different categories of motor imagery. There are also differences in the function of different brain regions, so the degree to which different channels of EEG signals discriminate between different categories of motor imagery varies. The ERD/ERS phenomenon shows that the frequency domains associated with motor imagery mainly concentrated in the alpha band (8–13 Hz) and the bate band (13–30 Hz), and the channels mainly concentrated in the motor areas of the parietal lobe.

Although these frequency domains and channels are the main frequency domains and channels for performing motor imagery classification, the study shows that motor imagery has individual variability and there is still a degree of variation in the specific frequency domains and channels of different subjects; on the other hand, these frequency domains and channels are only for the energy features in the synchronous mode. There is no clearer frequency and channel selection tendency for the distinction between NC and IC states in asynchronous mode as well as PLV features. For these reasons, it is necessary to perform feature screening on a larger scale.


(6) $$p_{n}=\sum_{i=1}^{n}a\,\left(m\right)\,p_{i}\cdot \ln\left(p_{i}\right)\,r_{n}^{m}$$

After music feature extraction for music, we successfully convert the music signal into a music feature signal, and the next step is to find the event points on the music feature signal. The music feature signal contains many mutations, but not all mutations can be regarded as feature event points because we use the ERP technique, so the feature event points should satisfy the characteristics of both mutation points and ERP events, therefore, we define the feature event points simply.

### Experimental Design of Interactive Electronic Music Piece Sentiment Classification

The energy signature reflects the physiological properties of motor imagery, but it is a single-channel signature that is more susceptible to the non-smoothness of the EEG signal. Our brain is a complex information processing center, and to process information more efficiently, the brain is divided into multiple functional areas, each of which is responsible for a different task; for example, image information processing is handled by the visual cortex located in the occipital lobe, and motor function is handled by the motor area located in the frontal lobe. The processing of complex tasks relies on the cooperation of multiple functional areas. The cooperation of multiple functional areas is carried out through information interaction, which is reflected in the scalp EEG as a dual-channel feature between channels (Mitrović, 2020). It has been shown that dual-channel features carry more information, reflect changes in brain state, and are more stable than single-channel features. This is advantageous for differentiating between controlled and uncontrolled states in asynchronous motor imagery. Common methods for analyzing dual-channel features include Granger causality, directed transition functions, Pearson correlation coefficients, phase synchronization, etc. Granger causality and directed transition functions reflect directed features, i.e., the features will differ with the order of the two channels. In the eyes of many people, empathy and compassion are similar concepts. They have the same roots and basically the same emotion generation and triggering. However, from a psychological point of view, the two are completely different. Compassion is like a bottle of fermented rice wine. Although it is also produced by the catalyzed fermentation of external interference factors, it does not take a long time. And empathy is like a bottle of high-year aging. It has a long time and a long and long process, and the temperament is completely different. The Pearson correlation coefficient and phase synchronization reflect undirected features, i.e., for a channel pair, the same result is obtained regardless of who comes first and who comes second in the calculation of the features.

**FIGURE 3:**
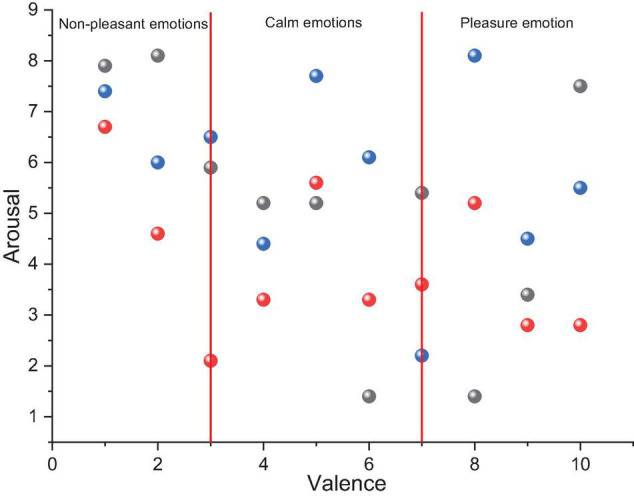
Emotional division map.

Sound is produced and propagated by the vibration of a sound source causing the same rhythmic vibration of the propagating medium, and different frequencies of sound have different senses of vibration. The physical phenomenon of vibration does not occur only in sound phenomena; vibration is a universal phenomenon that occurs in the universe and occurs frequently in the visual matter, and the use of sound vibrations can trigger a description of the form of vibration produced by visible matter (Tahıroğlu et al., 2020). Sound waves can be represented numerically and precisely by physical quantities such as frequency and amplitude, and through the perception of the extremely physical properties of the physical sound generation process, in physics sound waves are usually described and calculated mathematically for the commonality of their generation principles, i.e., a curved sonogram describing the periodic changes of sound energy at different moments experiencing different displacements utilizing a mathematical number axis, as shown in Figure 3. The sonogram can accurately show the numerical variation of physical quantities of sound waves such as frequency and amplitude with time and is important for analytical work on sound waves.

In the same experiment, the default parameter records may not fully satisfy the user’s needs, so the interface provides an interface to enable the user to store their own experiment information record file, which can be saved by clicking on it, or set to be saved automatically when the index is updated, and to complete the update of the index to that file path record at the same time. The stored data can also be analysis result data, each analysis result is marked with a timestamp, so the analysis result data can be directly displayed with the original data time match. The start and end times of data collection are recorded, and the data type of the file is recorded, with the data type taking on different values depending on the content of the experiment and the data stored. Offline analysis may be multiple times, by the index field to mark the number of times this processing results, to correspond to the offline processing of the analysis process records, to facilitate the comparison of experimental results.

**FIGURE 4:**
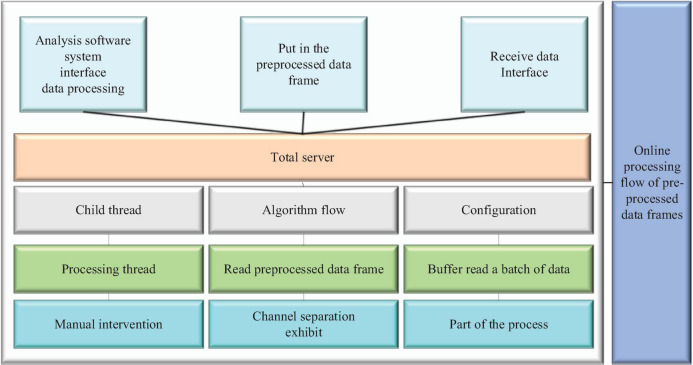
Online processing flow of pre-processed data frames.

According to the storage structure of index and data file separation and the mechanism of splitting the data storage by experiment and date to avoid oversized file storage, the approach used in this thesis achieves more efficient data retrieval. An example of the data retrieval process is shown in the figure below, first retrieve the experiment type and list all index file names of that type according to the experiment type. Then read the index file, the playback sub-interface displays the date with valid data recorded in the current index file, the user selects a date that needs to be played back, after selecting the interface will display the experimental records corresponding to that date, including the valid channel and the start and end time, according to the valid channel of the experimental records to select the channel number of the required playback, according to the listed start and end period to select the time, and finally locate the storage file. After locating the data file, read the data segment offset recorded in the corresponding index file, mark the valid period of the playback progress bar according to the start and end time of different data segments, and then read the data at the file offset corresponding to that time according to the progress bar position, as shown in Figure 4.

During the algorithm processing, the complexity of the set algorithm may be too high and the data in the buffer is not read in time. The mechanism of the buffer is that if the old data is not read in time for the same channel and the same frame number, it will be overwritten by the new data, and when the overwriting is done, the flag amount of data will be checked, and if it has not been read, it will be counted, and when this count exceeds a certain threshold (Calderón-Garrido et al., 2020). Music characteristic signal contains many mutations, but not all mutations can be regarded as characteristic event points. Because we adopt ERP technology, the characteristic event points should satisfy both the characteristics of mutation points and ERP events at the same time. The algorithm processing complexity is too high, resulting in data loss. The system parameter configuration includes local parameter configuration, acquisition system, and stimulation system parameter configuration. Since there is no hardware available for testing the stimulation system, only the first two are tested, which correspond to the local parameter column and acquisition system parameter column, respectively. After modifying the parameters in the corresponding parameter column, click the “Configure” button to issue the configuration command, the command successfully configured and prompted in the status bar; when the configuration command is issued in the case of network disconnection caused by disconnecting the network cable, the interface prompts “Configuration failed.” The interface prompts the normal connection logic and verifies the correctness of the parameter configuration function.

## Analysis of Results

### Multi-Brain Synergistic Brain-Computer Interface EEG Neurofeedback Results

**FIGURE 5:**
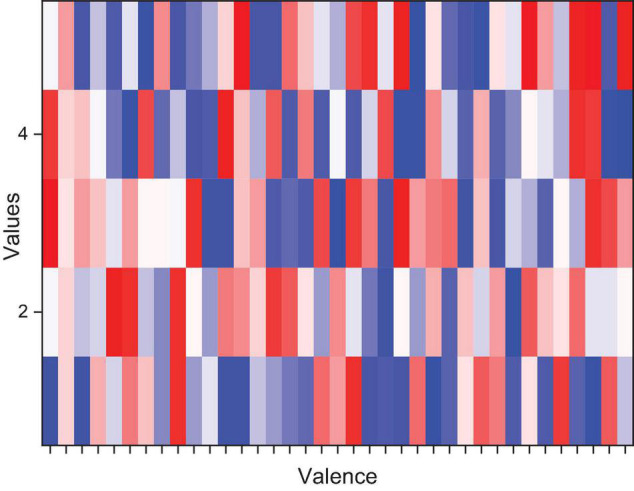
Object valence sentiment correlation coefficient.

A comparison of multiple feature combinations and learner combinations was attempted after completing the preprocessing and sentiment label modeling. Firstly, since the effect of the experimental subjects on the classification situation was not determined, the subjects with similar sentiment patterns were first determined by looking at the correlation coefficient matrix of the sentiment labels of the experimental subjects (32 people in total). As shown in Figure 5, a correlation coefficient matrix analysis was done for the 5 categorical labels of the 32 individuals in the Valence effective direction. Compared with the single-channel feature, the dual-channel feature carries more information, can better reflect the state of the brain, and has stronger stability. A dark red correlation coefficient of 1 represents a perfect correlation, while dark blue represents no linear relationship between the two. There are few red components in the whole graph (i.e., each person’s emotional perception of the same music varies widely) in which the 18 subjects with the closest emotional experience were selected 2, 3, 4, 8, 9, 11, 12, 13, 14, 15, 17, 18, 19, 20, 22, 24, 29, and 32 to verify by testing all subjects and selecting subjects for the validity of the EEG emotion classification.

**FIGURE 6:**
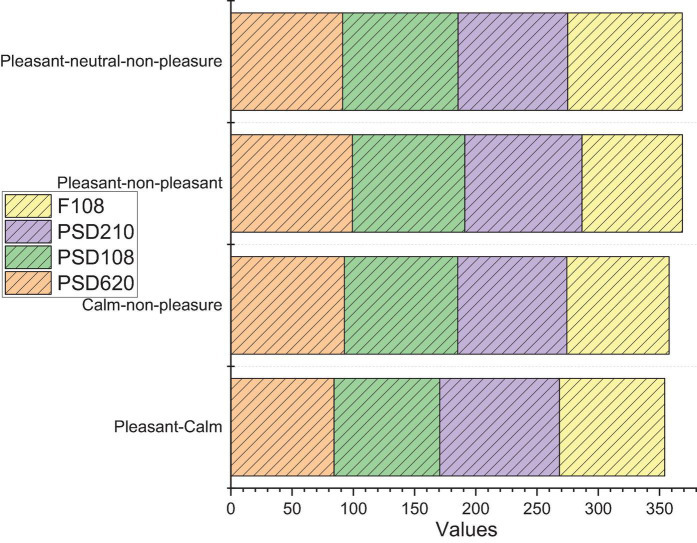
Classification results.

Compared to the pre-trained model on the DEAP training set itself, there is a decrease in the self-provided dataset. Compared to the model under PNN simultaneous training, the model under classification with SVM has average decrease incorrectness of 29.29% with Liking, which has always been accurate in classification, decreasing significantly. The overall correctness rate in the self-collected dataset is relatively lower. Of note is that Dominance has a significantly lower correct rate than several other labeled classifications. This may be partly due to the lack of a more uniform understanding of Dominance. On the other hand, compared to DEAP model predictions, the model under training using PNN neural networks may be relatively more consistent with the data distribution under DEAP, with reduced generalizability and the possibility of overfitting. There may be multiple offline analysis, and the number of processing results is marked by the index field to record the analysis process corresponding to offline processing to facilitate the comparison of experimental results. Next is the specific data of the experimental results. While the sample size of the self-collected training set is smaller and less likely to converge, this part of the test may also have differences in EEG equipment and various processing details at the same time. Therefore, attention needs to be paid to the explanation and filling of experimental labels, the debugging and processing of experimental EEG equipment, and the effects on EEG and emotion in different cultural backgrounds, different experiences, etc., as shown in Figure 6.

From the experimental results, we can see that the difference between the recognition effect of PSD620 and F108 is small, while the recognition rate of PSD108 obtained by dimensionality reduction of PSD620 is significantly lower than the above two feature sets. This indicates that we have achieved the purpose of dimensionality reduction based on the cognitive laws while ensuring the recognition rate. Subsequently, we performed sentiment recognition on dataset 2 using F108. Dataset 2 had 32 subjects watching 40 music videos, so there were 1,280 samples, and the same treatment was done for Dataset 1. A total of 15 electrodes in the frontal and central regions were selected for the DEAP database, with 6 sets of electrodes being paired electrodes, so the feature dimension of the DEAP database was again 108-dimensional features.

The main reasons for the low recognition rate obtained in this experiment are: the features extracted through this experiment are based on music-evoked emotion, and the DEAP database not only has music-evoked emotion factors, but also video-evoked emotion factors, so there are some problems in using the method of this experiment directly; secondly, as discussed before, the DEAP database has two channels of emotional stimuli, and there is a partial problem of mismatching the emotional content of the music videos, thus making the emotions evoked by the subjects not unique and thus resulting in low recognition rates in this paper. Brain cognitive mechanisms were investigated and emotions were classified using information based on the time and frequency domains of EEG signals. Brain activity in different affective states was first analyzed by brain topography to summarize the main active brain regions and frequency bands of emotion. Subsequently, the discovered cognitive laws were used in practical emotion recognition, and feature extraction was filtered and optimized to achieve the purpose of reducing feature dimensionality and improving recognition rate.

### Experimental Results of Emotional Classification of Interactive Electronic Music Pieces

**FIGURE 7:**
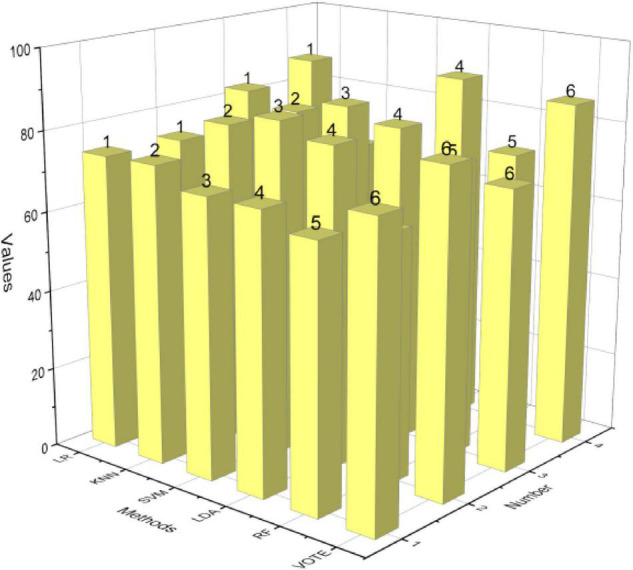
Taxonomy results.

After analyzing the variability of brain load in multiple states in asynchronous motor imagery, we verified the applicability of the brain load metric MWL to asynchronous motor imagery. Next, we investigate the effectiveness of MWL for the classification of asynchronous motor imagery, and we add MWL metrics to energy and PLV features of motor areas to investigate its enhancement of the classification performance of conventional features. We added MWL features to energy features, PLV features, and composite features for classification, and integrated the energy features with MWL features added with PLV features with MWL for learning classification, and used the same two-step classification method and direct triple classification method as in Chapter 3, and the average classification results for the seven subjects are shown in Figure 7.

The accuracy rate has dropped by 29.29% on average, and Liking, which has always been classified accurately, has dropped significantly. In the self-collected data set, the overall accuracy rate is relatively lower. The classification results from the two-step classifier show that for each of the motion area features, the addition of MWL features resulted in an improvement in accuracy on most of the classification algorithms. Among them, the one that improved on all six classifiers was the composite feature, with an improvement of 2.2–4.9%. The classification results from the direct tri-classification method show that for each of the motion area features, the addition of the MWL features resulted in an accuracy improvement on most of the classification algorithms. The highest average improvement for all 6 classifiers is the classification result for the integration learning of energy features with PLV features, with an average improvement of 3.35% and a maximum improvement of 7.3%. The classification result with the highest average improvement of 3.35% and the highest improvement of 7.3% for all 6 classifiers is the classification result of integrated learning of energy features with PLV features. This shows that the brain load metric has a boosting effect on the conventional features of an asynchronous motor imagery classification.

To verify the presence of elevated brain load from the uncontrolled to the controlled state, we investigated the variability of power spectral density in the theta and alpha bands in the controlled and uncontrolled states. From the brain topography, there was indeed a change in power spectral density consistent with elevated brain load. We then verified the significance of the MWL metric between the different states of asynchronous motor imagery, showing that for most subjects, there were significant differences both between the control and non-control states and between imagining the left hand and imagining the right hand and non-control states. Finally, we added MWL metrics to the regular energy and PLV features for classification, and the results showed boosts on most feature-classifier combinations, with the most significant boosts for composite features in the two-step classification method and integrated learning classification in the direct triple classification method. This suggests that brain load, a brain state feature, can improve feature stability in asynchronous motor imagery.

**FIGURE 8:**
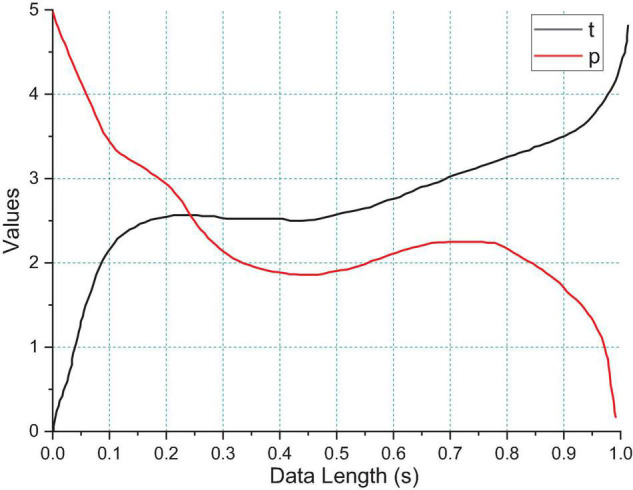
Results of paired *t*-test for difference in online recognition correctness for different data lengths.

The difference in recognition correctness between the two conditions in the pre-test and post-test experiments shown in Figure 8. The difference in online recognition correctness for different data lengths in the pre-test and post-test experiments statistically analyzed using a paired *t*-test. The results show that there is a significant difference in the difference in recognition rate between the two conditions in the pre-test and post-test experiments at data lengths of 0.6, 0.9, and 1 s. From the statistical results, the correct recognition rate of c SSVEP-BCI gradually increases with the increase of data length, but the difference of recognition rate between the two conditions becomes larger with the increase of data length in the pre-test experiment, and the difference of recognition rate between the two conditions stabilizes in the post-test experiment.

The highest recognition rate of the dichotomous task using brain network features for emotion classification reached 73.8%, which is higher than the recognition of emotion recognition based on time-frequency features. This indicates that our proposed dynamic brain network can effectively distinguish between different kinds of emotions Also the results of arousal-based emotion recognition are higher than those of pleasure-based emotion recognition are also consistent with our previous findings that brain network properties are more discriminative for differences in arousal. Among them, 6 groups of electrodes are pairs of electrodes, so the feature dimension of the DEAP database is also 108-dimensional features. From the classifiers, we can see that MLP and SVM perform the best, with near-identical recognition rates for both. This is exactly the opposite of the average clustering coefficient, which illustrates the dynamic balance of the brain. When the average clustering coefficient is elevated, it indicates that there is frequent information exchange between some brain regions and the surrounding brain regions, when decreasing the average path length between brain regions can improve the efficiency of information interaction between brain regions. This, in part, accounts for the self-regulatory nature of the brain. Similarly, LVHA emotions and LVLA emotions, the distinction between the two is more obvious. We perform feature extraction and optimization based on the previous findings; we optimize the traditional power spectral density-based feature extraction by cognitive laws to achieve feature dimensionality reduction while ensuring the recognition rate. We used brain networks as topological features of EEG signals for emotion classification and achieved an emotion recognition rate of 67.3% under four classifications, which exceeds the highest recognition rate available.

## Conclusion

We analyze the time-frequency characteristics of EEG signals, we construct the corresponding brain topography map by power spectral density and analyze the brain topography map, we find that the main brain regions associated with emotion evocation are central, frontal, and occipital regions and the main emotion-related EEG bands are delta band, theta band, alpha band, and gamma band. Ten subjects completed the game experiments, and the experimental results showed that all subjects were able to complete the game tasks better and achieve more efficient output. The experimental results showed that all subjects were able to complete the game task better and achieve more efficient output, proving the stability and reliability of the system and the effectiveness of the asynchronous classification algorithm. There seems to be a link between visual and auditory senses, which leads to a common audio-visual association. At this time, reducing the average path length of the brain section can improve the efficiency of the brain section information exchange. This explains the self-regulation of the brain to a certain extent. The phenomenon of audiovisual association provides the basis for the mapping transformation between sound and visual representation in this paper. Based on the analysis of the physiological and psychological causes of audiovisual association, various audiovisual mapping models are proposed in terms of people’s perception and aesthetics of sound, respectively. In the same interactive sound visualization work, one or more audiovisual mapping modes can be chosen to be applied. Such audiovisual mapping transformation patterns are based on the audiences’ audiovisual association phenomena, which are in line with the audiences’ perceptual and aesthetic perceptions of sound, and provide a basis for the subsequent research on visual representation methods.

## Data Availability Statement

The original contributions presented in the study are included in the article/supplementary material, further inquiries can be directed to the corresponding author.

## Author Contributions

ML: kualization and writing methodology, formal analysis, investigation resources, organized the database, and analyzed and interpreted the data.

## Conflict of Interest

The author declares that the research was conducted in the absence of any commercial or financial relationships that could be construed as a potential conflict of interest.

## Publisher’s Note

All claims expressed in this article are solely those of the authors and do not necessarily represent those of their affiliated organizations, or those of the publisher, the editors and the reviewers. Any product that may be evaluated in this article, or claim that may be made by its manufacturer, is not guaranteed or endorsed by the publisher.

## References

[B1] Biasutti M. Concina E. (2021). Online composition: strategies and processes during collaborative electroacoustic composition. *Br. J. Music Educ.* 38 58–73.

[B2] Calderón-Garrido D. Gustems-Carnicer J. Carrera X. (2020). Digital technologies in music subjects on primary teacher training degrees in Spain: teachers’ habits and profiles. *Int. J. Music Educ.* 38 613–624. 10.1177/0255761420954303

[B3] D’Agostino M. E. (2020). Reclaiming and preserving traditional music: aesthetics, ethics and technology. *Organ. Sound* 25 106–115. 10.1017/s1355771819000505

[B4] Dahan K. (2020). A temporal framework for electroacoustic music exploration. *Organ. Sound* 25 248–258. 10.1017/s1355771820000151

[B5] Farrell H. (2021). Considering dissemination. *Irish J. Technol. Enhanced Learn.* 6 14–21.

[B6] Görgün İ (2020). Exploring temporality in Horacio Vaggione’s compositional thought. *Organ. Sound* 25 168–178. 10.1017/s1355771820000072

[B7] Iverson J. (2019). *Electronic Inspirations: Technologies of the Cold War Musical Avant-Garde.* New York, NY: Oxford University Press.

[B8] Kerékfy M. (2020). “Zero point” the beginnings of György Ligeti’s Western career. *Stud. Musicol.* 60 103–114. 10.1556/6.2019.00005

[B9] Lakatos M. (2020). Sights and sounds of big data: Ryoji Ikeda’s immersive installations. *Acta Univ. Sapientiae Film Media Stud.* 18 109–129. 10.2478/ausfm-2020-0006

[B10] Malecki P. Piotrowska M. Sochaczewska K. Piotrowski S. (2020). Electronic music production in ambisonics-case study. *J. Audio Eng. Soc.* 68 87–94. 10.17743/jaes.2019.0048

[B11] Mitrović M. (2020). Transmedial (R) Evolution:{SinOsc. ar (400, 800, 0, 0.1) multisensory. experience}. play. *INSAM J. Contemp. Music Art Technol.* 1 60–76.

[B12] Redhead L. (2021). *Makis Solomos, From Music to Sound: The Emergence of Sound in 20th-and 21st-Century Music.* Abingdon: Routledge.

[B13] Romão J. (2020). Volker Müller & Co.: electronic music and sound engineering at the WDR. *Contemp. Music Rev.* 39 648–662.

[B14] Rossetti D. Antunes M. Manzolli J. (2020). Compositional procedures in electronic music and the emergence of time continuum. *Organ. Sound* 25 156–167.

[B15] Simurra I. Borges R. (2021). Analysis of Ligeti’s atmosphères by means of computational and symbolic resources. *Rev. Música* 21 369–394.

[B16] Smith B. E. Pacheco M. B. Khorosheva M. (2021). Emergent bilingual students and digital multimodal composition: a systematic review of research in secondary classrooms. *Read. Res. Q.* 56 33–52. 10.1002/rrq.298

[B17] Sofer D. (2020). Categorising electronic music. *Contemp. Music Rev.* 39 231–251.

[B18] Tahıroğlu K. Magnusson T. Parkinson A. Garrelfs I. Tanaka A. (2020). Digital musical instruments as probes: how computation changes the mode-of-being of musical instruments. *Organ. Sound* 25 64–74. 10.1017/s1355771819000475

[B19] Turchet L. West T. Wanderley M. M. (2021). Touching the audience: musical haptic wearables for augmented and participatory live music performances. *Pers. Ubiquitous Comput.* 25 749–769.

[B20] Wolffenbüttel D. C. R. Pellin H. (2020). Sound research and composition with everyday sounds. *Int. J. Dev. Res.* 10 34464–34471.

